# Analysis of UGT1A1*28 genotype and SN-38 pharmacokinetics for irinotecan-based chemotherapy in patients with advanced colorectal cancer: results from a multicenter, retrospective study in Shanghai

**DOI:** 10.1007/s00432-013-1480-7

**Published:** 2013-07-28

**Authors:** Xun Cai, Weiguo Cao, Honghua Ding, Tianshu Liu, Xinli Zhou, Mei Wang, Ming Zhong, Ziyi Zhao, Qing Xu, Liwei Wang

**Affiliations:** 1 Department of Oncology, Shanghai First People’s Hospital, Shanghai Jiaotong University, 100 Haining Road, Hongkou District, Shanghai, 200080 People’s Republic of China; 2 Department of Oncology, Ruijin Hospital, Medical School, Shanghai Jiaotong University, Shanghai, 200025 People’s Republic of China; 3 Department of Oncology, Zhongshan Hospital, Fudan University, Shanghai, 200032 People’s Republic of China; 4 Department of Oncology, Huashan Hospital, Fudan University, Shanghai, 200040 People’s Republic of China; 5 Department of Oncology, Changhai Hospital, Second Military University, Shanghai, 200433 People’s Republic of China; 6 Department of General Surgery, Renji Hospital, Medical School, Shanghai Jiaotong University, Shanghai, 200127 People’s Republic of China; 7 Department of General Surgery, Central Hospital in Jing’an District, Shanghai, 200040 People’s Republic of China; 8 Department of Oncology, Shanghai Tenth People’s Hospital, Shanghai Tongji University, Shanghai, 200072 People’s Republic of China

**Keywords:** Colonic neoplasms, Drug metabolism, Genetic polymorphism, Irinotecan, Uridine diphosphate glucuronosyl transferase

## Abstract

**Background:**

The UGT1A1*28 polymorphism, although closely linked with CPT-11-related adverse effects, cannot be used alone to guide individualized treatment decisions. However, CPT-11 dosage can be adjusted according to measured SN-38 pharmacokinetics. Our study is designed to investigate whether there is a relationship between SN-38 peak or valley concentrations and efficacy or adverse effects of CPT-11-based chemotherapy. We retrospectively studied 98 patients treated with advanced colorectal cancer in various UGT1A1*28 genotype groups (mainly (TA)_6_/(TA)_6_ and (TA)_6_/(TA)_7_ genotypes) treated with CPT-11 as first-line chemotherapy in Shanghai.

**Methods:**

One hundred and sixty-four advanced colorectal cancer patients were enrolled. To understand differences in genotype expression, the frequency of UGT1A1*28 thymine–adenine (TA) repeats in TATA box arrangement was assessed by PCR with genomic DNA extracted from peripheral blood. For ninety-eight cases with the (TA)_6_/(TA)_6_ and (TA)_6_/(TA)_7_ genotypes treated with CPT-11 as first-line chemotherapy, the plasma concentration of SN-38 was detected by HPLC 1.5 and 49 h after CPT-11 infusion. Efficacy and adverse effects were observed subsequently, and the relationship between SN-38 plasma concentration and efficacy or adverse effects within genotype groups, as well as differences in efficacy and adverse effects between (TA)_6_/(TA)_6_ and (TA)_6_/(TA)_7_ genotypes were analyzed statistically.

**Results:**

One hundred and fourteen patients (69.51 %) were identified with the (TA)_6_/(TA)_6_ genotype, forty-eight patients (29.27 %) with the (TA)_6_/(TA)_7_ genotype, and two patients (1.22 %) with the (TA)_7_/(TA)_7_ genotype. The average peak and valley concentrations of SN-38 after CPT-11 infusion and plasma bilirubin average levels before and after CPT-11 treatment in the (TA)_6_/(TA)_7_ genotype group were all higher than those in (TA)_6_/(TA)_6_ group, and the difference was statistically significant (*p* = 0.00). Stepwise regression analysis showed that SN-38 peak and valley concentration was correlated with PFS in the (TA)_6_/(TA)_6_ genotype. In the (TA)_6_/(TA)_7_ group, SN-38 peak concentration was correlated with CPT-11 starting dose and OS, valley concentration correlated with plasma bilirubin levels before CPT-11 treatment, delayed diarrhea, and OS. For the (TA)_6_/(TA)_6_ genotype, mPFS of the SN-38 peak concentration >43.2 ng/ml subgroup was significantly longer than that of ≤43.2 ng/ml subgroup (8.0 ± 0.35 vs. 6.5 ± 0.79 months, *χ* ^2^ = 17.18, *p* = 0.00) with a relatively high incidence of Grade I/II° myelosuppression; for the (TA)_6_/(TA)_7_ genotype, there was no significant difference in mOS between the SN-38 valley concentration >16.83 ng/ml and ≤16.83 subgroups (17.3 ± 0.45 vs. 18.8 ± 0.50 months, *χ* ^2^ = 1.38, *p* = 0.24), but the former had a higher incidence of Grade III/IV° mucositis and delayed diarrhea. For 2 (TA)_7_/(TA)_7_ cases, although 25 % dose reduction of CPT-11, which is calculated according to body surface area, Grade IV° bone marrow suppression and Grade III° delayed diarrhea still occurred after CPT-11 treatment, though both adverse effects resolved and did not recur again after a 50 % dose reduction.

**Conclusion:**

The (TA)_6_/(TA)_6_ genotype and (TA)_6_/(TA)_7_ genotype accounted for the most, and (TA)_7_/(TA)_7_ genotype only account for a very small portion of advanced colorectal cancer patients in Shanghai. For the (TA)_6_/(TA)_6_ genotype, CPT-11 dosage can be increased gradually to improve efficacy for patients with SN-38 peak concentration ≤43.2 ng/ml after CPT-11 infusion; and for (TA)_6_/(TA)_7_ genotype patients, CPT-11 dosage may be lowered appropriately to reduce serious adverse effects such as bone marrow suppression and delayed diarrhea without affecting the efficacy for those with SN-38 valley concentration >16.83 ng/ml. For (TA)_7_/(TA)_7_ genotype patients, adverse effects should be closely observed after treatment even if CPT-11 dosage has been reduced.

## Introduction

China has a high incidence of colorectal cancer due to a variety of factors including lifestyle changes with 400,000 newly diagnosed cases each year. Colorectal cancer is the second most common malignant tumor in Shanghai, with incidence increasing by 3.67 times since the early 1970s. Between 40 and 70 % patients will experience postoperative recurrence and metastasis following radical surgery for early-stage disease. CPT-11-based chemotherapy has been supported by the research as a standard first-line treatment for advanced colorectal cancer. However, Grade III/IV° bone marrow suppression or delayed diarrhea caused by CPT-11 may occur in some patients during or after treatment, which leads to treatment delays and affect patients’ quality of life (Douillard et al. 2000; Innocenti et al. 2004). Many studies have confirmed that UGT1A1 is the main metabolic enzyme that inactivates SN-38, the active product of CPT-11, into SN-38G. Ethnic differences exist in UGT1A1 enzyme activity and gene polymorphisms (Kaniwa et al. 2005), which are closely related to CPT-11 adverse effects (Innocenti et al. 2004; Massacesi et al. 2006) but they are uncertain with efficacy (Shulman et al. 2011; Liu et al. 2008). In addition, studies have found that AUC_SN-38_ or AUC_SN-38G_/AUC_SN-38_ is associated with neutropenia after CPT-11 treatment (Hirose et al. 2012; Canal et al. 1996), which indicate that CPT-11 or SN-38 pharmacokinetics is as important as UGT1A1 gene polymorphism analysis in predicting CPT-11-related adverse effects, even in dose individualization sets. Presently, data are limited about the role of UGT1A1*28 genotype and SN-38 pharmacokinetics in predicting CPT-11 efficacy and adverse effects, so our study reviewed the plasma SN-38 peak and valley concentration, treatment efficacy, and adverse effects in 98 cases with (TA)_6_/(TA)_6_ and (TA)_6_/(TA)_7_ genotypes who were treated with CPT-11 as first-line chemotherapy, in order to determine whether there is a relationship between plasma SN-38 peak and valley concentration and efficacy or adverse effects in patients with different genotypes, and provide theoretical basis for CPT-11 treatment individualization based on UGT1A1*28 genotype in combination with SN-38 pharmacokinetics analysis.

## Materials and methods

### Patient eligibility

A total of 164 hospitalized patients with local advanced and metastatic colorectal cancer were eligible for the study. Disease was confirmed with pathological and imaging data in Zhongshan Hospital, Huashan Hospital affiliated with Fudan University, Ruijin hospital, Renji hospital affiliated with Shanghai Jiaotong University Medical College, Changhai hospital affiliated with the Second Military University, Shanghai No. 1 and No. 10 People’s Hospital, and the Central Hospital of Jing’an District from April 2010 to January 2012. Of the eligible patients, 117 were males and 47 were females, aged from 26 to 75 years with a median age of 60 years, and included 100 cases of colon cancer and 64 cases of rectal cancer. Twenty cases had stage IIIb disease and 144 cases had stage IV disease according to the AJCC Cancer Staging standard (6th edition). Inclusion criteria were as follows: ECOG physical status scores from 0 to 2, with measurable disease not treated and life expectancy of ≥3 months; of all the 164 cases, 98 cases were assigned to receive a modified FOLFIRI chemotherapy regimen for at least 4 cycles as first-line palliative chemotherapy after excluding chemotherapy contraindications and obtaining informed consent. Plasma bilirubin and transaminase level could not exceed 1.5 times and 5 times the normal upper limit for patients with liver metastases. Exclusion criteria such as pregnant or lactating women, patients with complete or incomplete intestinal obstruction or with a history of chronic enteritis or extensive bowel resection, or with central nervous system metastases, having been seriously allergic to drugs and its excipients used in the treatment, evaluable lesions which had received radiotherapy or other treatments, vital organ dysfunction, history of other malignancies (cervical carcinoma in situ or skin basal cell carcinoma excluded) and poor compliance.

About 2 ml of peripheral blood sample (anticoagulated with EDTA) was obtained from each patient before chemotherapy for UGT1A1*28 genotype analysis, and then the patients received FOLFIRI chemotherapy 1.5-h infusion of CPT-11 (180 mg/m^2^) and 2-h infusion of leucovorin (400 mg/m^2^), followed by 5-fluorouracil bolus (400 mg/m^2^) on day 1 and a 46-h continuous infusion (2,400 mg/m^2^) for each cycle, repeated every 2 weeks. About 2 ml of peripheral blood sample was taken 1.5 and 49 h after CPT-11 infusion for SN-38 plasma concentration detection by HPLC (Hirose et al. 2012; Schoemaker et al. 2003), and CPT-11 dosage was lowered by 20–25 % in case of Grade III° adverse effects.

### UGT1A1*28 genotype analysis

Genomic DNA was extracted with DNA extraction kit (Qiagen Inc, Valencia, CA, USA) for PCR amplification. Prime sequences were designed as follows: Upstream: 5′-TCCCTGCTACCTTTGTGGAC-3′, downstream: 5′-AGCAGGCCCAGGACAAGT-3′. The PCR mix was (25 μl): 2 μl of 10× PCR buffer with 15 mM MgCl_2_, 2 μl of dNTP (2.5 mM), 1 μl of Primers (10 μm), 1 μl of DNA templates, 0.2 μl of DNA Taq polymerase (5U/μl), and 18.8 μl of ddH_2_O. Amplification procedure was as follows: a initial denaturation step at 94 °C for 5 min; followed by 40 cycles of denaturation (94 °C for 15 s), annealing (55 °C for 25 s) and extension (72 °C for 50 s), and finally, extension at 72 °C for 7 min. If the PCR product band is clear by electrophoresis, take 5 μl of eligible specimen, mixed by adding 2 μl SAP, kept at 37 °C for 60 min and 80 °C for 15 min, and then saved at 4 °C. Took 3 μl positive PCR hydrolysates, 1 μl sequencing reagent (bigdye), and 2 μl sequencing primer for PCR amplification: initial denaturation at 96 °C for 1 min, followed by 25 cycles of denaturation (96 °C for 10 s), annealing (50 °C for 5 s) and extension (60 °C for 4 min), and then stored at 4 °C. The sequencing product was sequenced in DNA sequencing instrument (ABI-373, PE corp., USA) after being purified. Sequencing results were displayed and analyzed with GeneMapper software.

### SN-38 plasma concentration detection

SN-38 and IS (acetone) were dissolved with dimethyl sulfoxide into 1.0 mg/ml stock solution stored at −80 °C. SN-38 reference standard plasma was prepared at a concentration range of 2–500 ng/ml with blank plasma. We then took 200 μl of the mentioned plasma, vortexed with acetonitrile (100 ng/ml) for 1 min, and centrifuged at 9,500 rpm for 10 min, and then tested after taking 200 μl supernatant vortexed with 100 μl hydrochloric acid (1 mol/L) for 30 s. The standard curve and linear regression were prepared by taking the ratio of peak area of SN-38 and IS as the vertical axis. Chromatographic condition: Waters^®^Nova-Pak C18 Guard column (20 mm × 3.9 mm, 5 μm of particle diameter); mobile phase: disodium hydrogen phosphate (0.05 mol/L, pH = 4.0, containing 0.05 mol/L 1-heptanesulfonic acid)-acetonitrile (75:25, v/v). Testing condition: flow rate of 1 ml/min, column temperature at 25 °C, excitation wavelength at 370 nm, and emission wavelength at 470 nm. The retention time for SN-38 and IS was 15.4 and 17.8 min under the experimental condition.

### Evaluation criteria and follow-up

The first efficacy follow-up was performed for all patients by imaging scans after 3 cycles of chemotherapy, and partial remission required reconfirmation at least 4 weeks after initial assessment. Re-evaluation was to be done every 3 cycles until disease progression and patients to be followed for overall survival every 3 months. Patients could receive other chemotherapy or best supportive care after disease progression, but not include targeted therapy. The median follow-up was 16 month. Tumor efficacy was evaluated using RECIST, and toxicity was graded according to CTCAE version 3.0.

### Statistical analyses

Data were expressed as mean ± standard deviation. Prognostic factors such as mPFS and mOS were calculated using the Kaplan–Meier method, and survival differences were analyzed by log rank test. A *t* test was used to compare the difference between the groups and a chi-square test to compare count data. Degree of adverse effects, plasma concentration, and efficacy was accessed by variance analysis. All values were two-sided, and statistical significance was accepted at the *p* < 0.05 level. SPSS version 16.0 software (SPSS Inc., USA) was used for all statistical analyses.

## Results

### UGT1A1 polymorphism for patients with advanced colorectal cancer in Shanghai

**Fig. 1:**
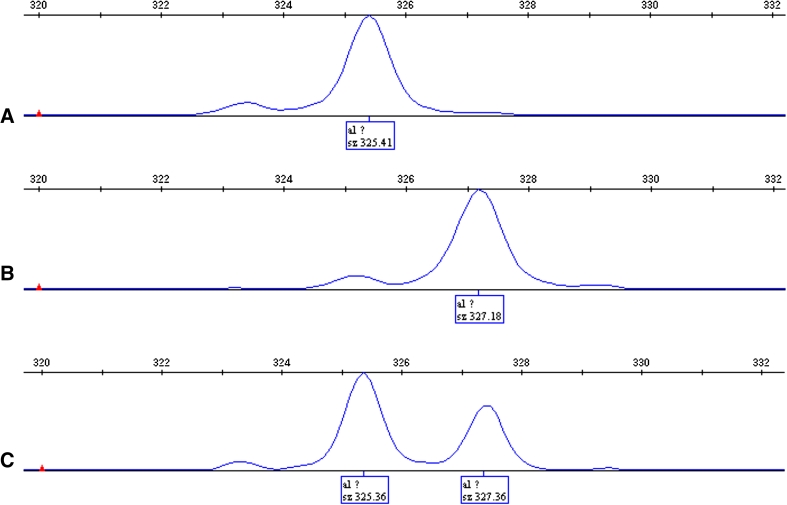
Sequencing results for (TA)_6_/(TA)_6_, (TA)_6_/(TA)_7,_ and (TA)_7_/(TA)_7_ genotypes with GeneMapper software. **a** (TA)_6_/(TA)_6_ genotype, **b** (TA)_6_/(TA)_7_ genotype, **c**: (TA)_7_/(TA)_7_ genotype

One hundred and fourteen patients (69.51 %) were identified with the (TA)_6_/(TA)_6_ genotype, forty-eight patients (29.27 %) with the (TA)_6_/(TA)_7_ genotype, and two patients (1.22 %) with the (TA)_7_/(TA)_7_ genotype (for sequencing results, see Fig. 1), and there were no statistical differences in age, gender, ECOG performance score, primary tumor site, TMN staging, and chemotherapy order between (TA)_6_/(TA)_6_ and (TA)_6_/(TA)_7_ groups except for CPT-11 dosage reduction in the (TA)_6_/(TA)_7_ group (see Table 1).

**Table 1:** Comparison of clinical characteristics between (TA)_6_/(TA)_6_ and (TA)_6_/(TA)_7_ groups

Clinical characteristics	6/6 genotype (*n* = 114)	6/7 genotype (*n* = 48)	*F*	*P*
ECOG performance score			2.34	0.27
0′	34	10		
1′	80	38		
Gender			1.87	0.31
Male	82	34		
Female	32	14		
Age (year)	57.54 ± 10.38	58.35 ± 10.06	0.21	0.65
Primary tumor site			0.66	0.50
Colon	66	34		
Rectum	48	14		
TMN staging			3.86	0.19
IIIb	12	8		
IV	102	40		
Chemotherapy order			0.42	0.58
First line	74	24		
Second line	40	24		
CPT-11 starting dosage (mg)	297.72 ± 35.30	299.17 ± 43.46	0.05	0.83
CPT-11 dosage reduction			23.67	0.00
Yes	8	17		
No	106	31		

### SN-38 peak and valley concentration after CPT-11 infusion and plasma bilirubin level before and after treatment for (TA)_6_/(TA)_6_ and (TA)_6_/(TA)_7_ groups treated with first-line chemotherapy

**Fig. 2:**
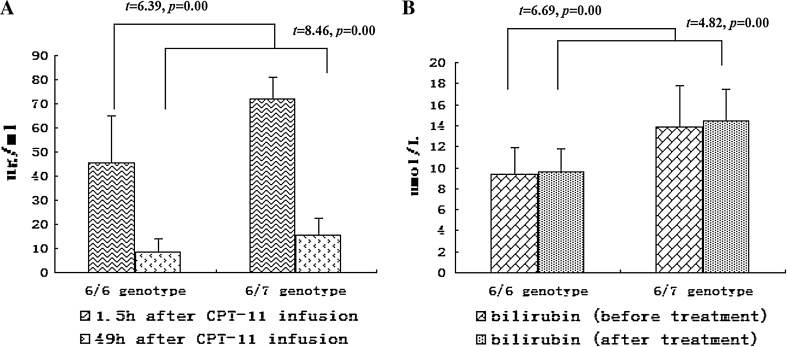
Comparison of SN-38 peak or valley concentrations after CPT-11 infusion and plasma bilirubin levels before and after CPT-11 treatment between (TA)_6_/(TA)_6_ and (TA)_6_/(TA)_7_ genotypes. **a** SN-38 peak and valley concentrations were 45.57 ± 19.38 and 8.67 ± 5.45 ng/ml for (TA)_6_/(TA)_6_ genotype, and 71.80 ± 9.15 and 15.39 ± 7.25 ng/ml for (TA)_6_/(TA)_7_ genotype after CPT-11 infusion, which were lower than the former with significant difference (*t* = 6.39, *p* = 0.00; *t* = 4.82, *p* = 0.00). **b** Plasma bilirubin level was 9.42 ± 2.43 and 9.56 ± 2.26 μmol/L for (TA)_6_/(TA)_6_ genotype, and 13.91 ± 3.90 and 14.44 ± 2.99 μmol/L for (TA)_6_/(TA)_7_ genotype before and after treatment with significant difference between genotypes (*t* = 6.69, *p* = 0.00; *t* = 8.46, *p* = 0.0) but without significant difference before and after treatment within genotype (*t* = 0.35, *p* = 0.72; *t* = 0.53, *p* = 0.60)

SN-38 average peak and valley concentration after CPT-11 infusion was 45.57 ± 19.38 and 8.67 ± 5.45 ng/ml, respectively, for the (TA)_6_/(TA)_6_ genotype group, and 71.80 ± 9.15 and 15.39 ± 7.25 ng/ml for the (TA)_6_/(TA)_7_ genotype, and the difference was statistically significant (*t* = 6.39, *p* = 0.00; *t* = 4.82, *p* = 0.00). Plasma bilirubin levels were 9.42 ± 2.43 and 9.56 ± 2.26 μmol/L for (TA)_6_/(TA)_6_ group, and 13.91 ± 3.90 and 14.44 ± 2.99 μmol/L for (TA)_6_/(TA)_7_ group before and after treatment, respectively, with significant difference between the groups (*t* = 6.69, *p* = 0.00; *t* = 8.46, *p* = 0.0) but without significant difference before and after treatment within groups (*t* = 0.35, *p* = 0.72; *t* = 0.53, *p* = 0.60), see Fig. 2.

### Stepwise regression analysis between SN-38 peak and valley concentration and efficacy, adverse effects

Taking SN-38 peak and valley concentration as dependent variables, and adverse reactions, short-term effect, PFS, and OS as independent variables, stepwise regression analysis showed that, in the (TA)_6_/(TA)_6_ group, SN-38 peak and valley concentration was related to PFS, but in the (TA)_6_/(TA)_7_ genotype, SN-38 peak concentration was related to CPT-11 starting dose and OS, and the valley concentration was related to plasma bilirubin levels before CPT-11 treatment, delayed diarrhea incidence, and OS (regression equation coefficients are shown in Table 2).

**Table 2:** Stepwise regression of SN-38 peak, valley concentrations with efficacy, and adverse effects

	Unstandardized coefficients		Standardized coefficients	*t*	*p*
	B	SE	Beta		
(TA)_6_/(TA)_6_ genotype					
SN-38 peak concentration					
Constant	−9.53	7.47		−1.28	0.21
PFS	8.48	1.12	0.67	7.58	0.00
SN-38 valley concentration					
Constant	−3.28	2.42		−1.36	0.18
PFS	1.84	0.36	0.51	5.08	0.00
(TA)_6_/(TA)_7_ genotype					
SN-38 peak concentration					
Constant	17.78	9.70		1.83	0.08
CPT-11 starting dosage	0.08	0.03	0.45	2.86	0.01
OS	1.78	0.64	0.44	2.80	0.01
SN-38 valley concentration					
Constant	−5.93	6.21		−0.95	0.35
Delayed diarrhea	3.64	1.05	0.47	3.46	0.00
OS	1.56	0.43	0.49	3.60	0.00
Bilirubin level before treatment	−0.70	0.25	−0.38	−2.82	0.01

### Analysis of SN-38 peak, valley concentration correlation with efficacy and adverse effects of the subgroups in (TA)_6_/(TA)_6_ and (TA)_6_/(TA)_7_ genotypes

**Fig. 3:**
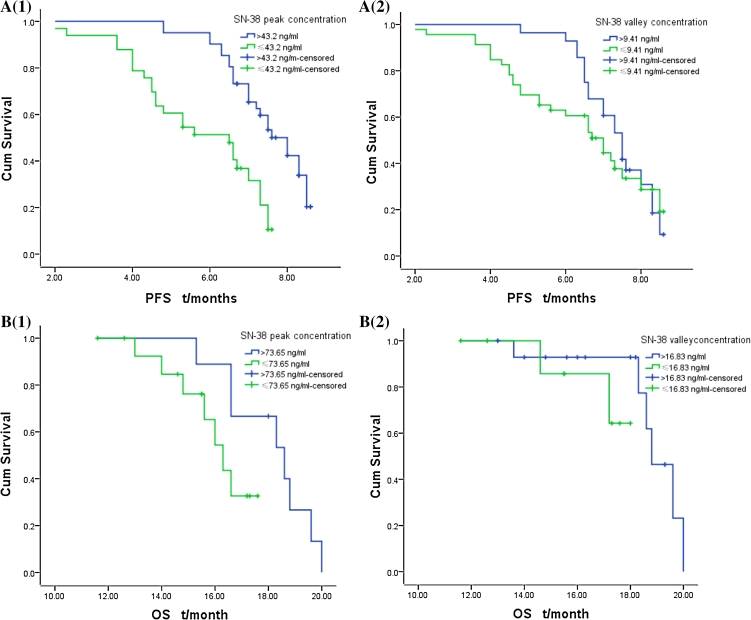
mPFS and mOS of the subgroups in (TA)_6_/(TA)_6_ and (TA)_6_/(TA)_7_ genotypes **A** in (TA)_6_/(TA)_6_ genotype, mPFS of SN-38 peak concentration >43.2, ≤43.2 ng/ml subgroup: 8.0 ± 0.35 versus 6.5 ± 0.79 months, *χ* ^2^ = 17.18, *p* = 0.00 (**A1**); mPFS of SN-38 trough concentration >9.41, ≤9.41 ng/ml subgroup in (TA)_6_/(TA)_6_ genotype: 7.5 ± 0.25 versus 7.0 ± 0.49 months, *χ* ^2^ = 0.75, *p* = 0.39 (**A2**)

According to the corrected predictive values and standard deviations of SN-38 peak and valley concentration (45.49 ± 2.29 and 8.67 ± 0.74 ng/ml; 71.58 ± 2.07 and 15.25 ± 1.58 ng/ml), the (TA)_6_/(TA)_6_ genotype was divided into subgroups for the analysis by peak and valley concentration >43.2 or ≤43.2 ng/ml and >9.41 or ≤9.41 ng/ml, respectively. The (TA)_6_/(TA)_7_ genotype was divided similarly with boundaries at >73.65 or ≤73.65 ng/ml and >16.83 or ≤16.83 ng/ml, respectively. We found that mPFS for the SN-38 peak concentration >43.2 ng/ml subgroup was significantly higher than that for the ≤43.2 ng/ml subgroup (8.0 ± 0.35 vs. 6.5 ± 0.79 months, *χ* ^2^ = 17.18, *p* = 0.00) only with a relatively high incidence of Grade I/II° myelosuppression in the former, but there was no significant difference in mPFS between the >9.41 and ≤9.41 ng/ml subgroups (7.5 ± 0.25 vs. 7.0 ± 0.49 months, *χ* ^2^ = 0.75, *p* = 0.39) for the (TA)_6_/(TA)_6_ genotype; no significant difference was found in mOS between SN-38 peak concentration >73.65, ≤73.65 ng/ml subgroups and valley concentration >16.83 ng/ml, ≤16.83 subgroups (18.6 ± 1.30 vs. 16.3 ± 0.51 months, *χ* ^2^ = 2.83, *p* = 0.09; 17.3 ± 0.45 vs. 18.8 ± 0.50 months, *χ* ^2^ = 1.38, *p* = 0.24), but the >16.83 ng/ml subgroup had a higher incidence of Grade III/IV° mucositis and delayed diarrhea than ≤16.83 subgroup (*F* = 5.58, *p* = 0.03; *F* = 19.60, *p* = 0.00, see Fig. 3; Table 3) for the (TA)_6_/(TA)_7_ genotype.

**Table 3:** Adverse effects of each subgroup with different SN-38 peak concentration and valley concentration for (TA)_6_/(TA)_6_ and (TA)_6_/(TA)_7_ genotype

Adverse effects in (TA)_6_/(TA)_6_ genotype		SN-38 peak concentration (ng/ml)		*F*	*p*	SN-38 trough concentration (ng/ml)		*F*	*p*
		>43.2 (*n* = 41)	≤43.2 (*n* = 33)			>9.41 (*n* = 28)	≤9.41 (*n* = 46)		
A (%)	I/II°	28/41 (68.29)	23/33 (69.70)	1.34	0.72	19/28 (67.86)	32/46 (69.57)	1.30	0.72
	III/IV°	0/41 (0)	1/33 (3.03)			0/28 (0)	1/46 (2.17)		
B (%)	I/II°	7/41 (17.07)	4/33 (12.12)	5.63	0.02	4/28 (14.29)	7/46 (15.22)	3.44	0.07
	III/IV°	0/41 (0)	0/33 (0)			0/28 (0)	0/46 (0)		
C (%)	I/II°	26/41 (63.41)	24/33 (72.73)	1.16	0.29	18/28 (64.29)	32/46 (69.57)	0.12	0.73
	III/IV°	1/41 (2.44)	0/33 (0)			0/28 (0)	1/46 (2.17)		
D (%)	I/II°	11/41 (26.83)	16/33 (48.49)	0.06	0.82	6/28 (21.43)	21/46 (45.65)	0.59	0.45
	III/IV°	0/41 (0)	0/33 (0)			0/28 (0)	0/46 (0)		
E (%)	I/II°	4/41 (9.76)	1/33 (3.03)	1.30	0.26	2/28 (7.14)	7/46 (15.22)	0.01	0.92
	III/IV°	0/41 (0)	0/33 (0)			0/28 (0)	0/46 (0)		

Adverse effects in (TA)_6_/(TA)_7_ genotype		SN-38 peak concentration (ng/ml)		*F*	*p*	SN-38 trough concentration (ng/ml)		*F*	*p*
		>73.65 (*n* = 9)	≤73.65 (*n* = 15)			>16.83 (*n* = 8)	≤16.83 (*n* = 16)		
A (%)	I/II°	3/9 (33.33)	9/15 (60.0)	0.28	0.60	2/8 (25.0)	10/16 (62.50)	3.79	0.06
	III/IV°	5/9 (55.56)	5/15 (33.33)			6/8 (75.0)	4/16 (25.0)		
B (%)	I/II°	2/9 (22.22)	5/15 (33.33)	0.31	0.58	5/8 (62.50)	2/16 (12.50)	5.58	0.03
	III/IV°	0/9 (0)	0/15 (0)			0/8 (0)	0/16 (0)		
C (%)	I/II°	3/9 (33.33)	9/15 (60.0)	1.24	0.28	5/8 (62.50)	7/16 (439.75)	1.24	0.28
	III/IV°	0/9 (0)	0/15 (0)			0/8 (0)	0/16 (0)		
D (%)	I/II°	7/9 (77.78)	10/15 (66.67)	1.05	0.32	5/8 (62.50)	11/16 (68.75)	19.60	0.00
	III/IV°	1/9 (11.11)	2/15 (13.33)			3/8 (37.50)	0/16 (0)		
E (%)	I/II°	0/9 (0)	4/15 (26.67)	3.0	0.10	1/8 (12.50)	3/16 (18.75)	0.30	0.59
	III/IV°	0/9 (0)	0/15 (0)			0/8 (0)	0/16 (0)		

A: bone marrow suppression; B: mucositis; C: nausea and vomiting; D: delayed diarrhea; E: liver function injury

### SN-38 peak, valley concentration, plasma bilirubin, and adverse effects for (TA)_7_/(TA)_7_ genotype

SN-38 average peak and valley concentration after CPT-11 infusion were 113.4 and 33.18 ng/ml, and the average plasma bilirubin levels before and after treatment were 20.9 and 24.1 μmol/L. Although 25 % dose reduction of CPT-11, which is calculated according to the body surface area, Grade IV° bone marrow suppression and Grade III° delayed diarrhea still occurred after CPT-11 treatment in 2 (TA)_7_/(TA)_7_ cases, both adverse effects resolved and did not recur after a 50 % dose reduction.

## Discussion

Clinical administration dosage is generally calculated according to the body surface area or weight presently (Reilly and Workman 1993), which is the group average dose, but in fact, only a part of drugs with low toxicity and conventional administration, which doses calculated as above, may get satisfactory effects. The plasma concentration may be affected by drug absorption, distribution, metabolism, and excretion, even small changes in plasma concentration may cause efficacy differences and lead to serious adverse effects. Anti-tumor therapy is entering the era of individualized treatment, through the use of biological markers, drug metabolism genes, or pharmacokinetics detection, etc. The purpose of individualized treatment for advanced colorectal cancer is to improve efficacy and/or to avoid and reduce the incidence of adverse effects (Gamelin et al. 2008; Ychou et al. 2003; Fety et al. 1998; Kleibl et al. 2009; Van Kuilenburg et al. 2002; Chang et al. 2009; Liu et al. 2011). CPT-11 is metabolized to its active product SN-38 by hCES in vivo and mainly inactivated to SN-38G by UGT1A1. It is generally believed that SN-38 determines activity and toxicity; however, most studies have not found a correlation between single-nucleotide polymorphisms and enzyme activity in UGT1A1 (Wu et al. 2004; Charasson et al. 2004), However, the UGT1A1 gene polymorphism can cause decline or absence of enzyme activity. A number of studies have indicated that the UGT1A1*28 homozygous genotype [(TA)_7_/(TA)_7_ genotype] may lead to weakening of SN-38 glucuronidation, which is an important risk factor for CPT-11-associated adverse effects such as delayed diarrhea and myelosuppression (Marcuello et al. 2004; Iyer et al. 2002; Paoluzzi et al. 2004). Our results showed that (TA)_6_/(TA)_6_ and (TA)_6_/(TA)_7_ genotypes accounted for 69.51 and 29.27 % of patients, respectively, and the (TA)_7_/(TA)_7_ genotype only for 1.22 % of the 164 enrolled patients in Shanghai, and that genotype had no association with age, gender, ECOG performance score, primary tumor site, TMN staging, or chemotherapy background, which was similar to other domestic studies (Yang et al. 2009; Zhang et al. 2007), but the proportion was lower for (TA)_6_/(TA)_6_ and (TA)_6_/(TA)_7_ genotypes than foreign studies (Iyer et al. 2002; Côté et al. 2007; Toffoli et al. 2006; Braun et al. 2008); it is also explained that CPT-11-related adverse effects are lower in Chinese than in foreigners.

Although the UGT1A1*28 heterozygous genotype [(TA)_6_/(TA)_7_ genotype] can lead to decrease in UGT1A1 activity, gene polymorphism analysis alone cannot be used to direct individualized treatment of CPT-11 because changes also occur in enzyme activity for this genotype with individual difference. Pharmacokinetic studies have shown that (TA)_7_/(TA)_7_ genotype has higher AUC_SN-38_ than (TA)_6_/(TA)_6_ and (TA)_6_/(TA)_7_ genotypes (Iyer et al. 2002), with CPT-11 reaching peak concentration 1.5 h after infusion, terminal half-life of 10.8 h, and fall to valley level of about 30 ng/ml 25.5 h roughly after infusion (Sumiyoshi et al. 1995); in addition, 5-fluorouracil dose not change CPT-11 metabolism in vivo in a certain range of dosage (Ducreux et al. 1999; Saltz et al. 1996). These results can help to give guidance on CPT-11 individualized treatment by plasma concentration detection. Despite (TA)_6_/(TA)_6_ and (TA)_6_/(TA)_7_ genotypes accounting for most cases, there are no detailed reports before about the differences in terms of efficacy and adverse effects after first-line treatment with CPT-11. Our study found that the average peak and valley concentrations of SN-38 in (TA)_6_/(TA)_6_ genotype were higher than those in (TA)_6_/(TA)_7_ genotype after CPT-11 infusion; although average plasma bilirubin was at normal levels in both genotypes, the levels in (TA)_6_/(TA)_7_ genotype are also higher than that in (TA)_6_/(TA)_6_ genotype before and after treatment with significant difference, which was consistent with the findings of Rouits et al. (2008). The cause for this phenomenon is that bilirubin and SN-38 are both substrates of UGT1A1, and abnormality in bilirubin glucuronidation leaves, bilirubin level elevated when UGT1A1 activity decreases, while CPT-11 metabolism may also be abnormal because of the substrate competition, which leads to elevation of SN-38 concentration.

Stepwise regression analysis showed that SN-38 average peak and valley concentration were associated with PFS in the (TA)_6_/(TA)_6_ genotype; however, SN-38 peak concentration was associated with CPT-11 starting dosage and OS, and valley concentration was associated with plasma bilirubin levels before CPT-11 treatment, delayed diarrhea, and OS in (TA)_6_/(TA)_7_ genotype. So we divided the two genotypes into four subgroups according to the upper and lower limit of the corrected predictive values and standard deviations for SN-38 average peak and valley concentrations, in order to analyze the relationship between plasma concentration and efficacy or adverse effects in the subgroups. The results showed that the mPFS of the SN-38 peak concentration >43.2 ng/ml subgroup was significantly higher than that of ≤43.2 ng/ml subgroup with a relatively high incidence of Grade I/II° myelosuppression in the (TA)_6_/(TA)_6_ genotype, while there was no significant difference in mOS between SN-38 valley concentration >16.83 ng/ml and ≤16.83 subgroups, but the former had a higher incidence of Grade III/IV° mucositis and delayed diarrhea in (TA)_6_/(TA)_7_ genotype. This suggested that CPT-11 dosage can be gradually increased to improve treatment efficacy in the SN-38 peak concentration ≤43.2 ng/ml subgroup after CPT-11 infusion without serious adverse effects for patients with the (TA)_6_/(TA)_6_ genotype (in our study, 55.41 %(41/74) of (TA)_6_/(TA)_7_ genotype with SN-38 peak concentration ≤43.2 ng/ml, which means more than half of the patients require dose adjustment), while CPT-11 dosage may be appropriately lowered to reduce the incidence of serious adverse effects without affecting the efficacy in the SN-38 valley concentration >16.83 ng/ml subgroup for the (TA)_6_/(TA)_7_ genotype. SN-38 peak and valley concentration and plasma bilirubin level in the (TA)_7_/(TA)_7_ genotype were significantly higher than that in the (TA)_6_/(TA)_7_ genotype, while the incidence of serious adverse effects was consistent with other studies (Marcuello et al. 2004; Iyer et al. 2002a, b; Paoluzzi et al. 2004).

## Conclusions

In summary, our study suggests that the UGT1A1*28 (TA)_6_/(TA)_6_ and (TA)_6_/(TA)_7_ genotypes account for most patients, while the homozygous mutant genotype only accounts for a very small portion of patients with advanced colorectal cancer in Shanghai. For the (TA)_6_/(TA)_6_ and (TA)_6_/(TA)_7_ genotypes, UGT1A1*28 gene polymorphism in combination with SN-38 pharmacokinetics analysis provides a good theoretical basis for CPT-11 individualized treatment; however, it should be verified by further expanding sample sizes and repeated determination of SN-38 plasma concentration after dosage adjustment, in order to improve efficacy and meanwhile avoid or reduce serious adverse effects. For the (TA)_7_/(TA)_7_ genotype, despite the reduction in initial treatment dose, further adjustment of CPT-11 dosage still should be done according to adverse effects after treatment.

## Abbreviations

HPLC: High-performance liquid chromatography
PFS: Progression-free survival
mPFS: Median PFS
OS: Overall survival
mOS: Median OS
UGT1A1: Uridine diphosphate glucuronosyl transferase 1A1
SN-38: 7-ethyl-10-hydroxycamptothecin
SN-38G: SN-38 glucuronide
AUC_SN-38_: Area under the curve of SN-38
AUC_SN-38G_/AUC_SN-38_: The ratio of area under the curve of SN-38G and SN-38
SAP: Shrimp alkaline phosphatase
IS: Internal standard
RECIST: The response evaluation criteria in solid tumors
CTCAE: National Cancer Institute common terminology criteria for adverse events
hCES: Human carboxylesterase
